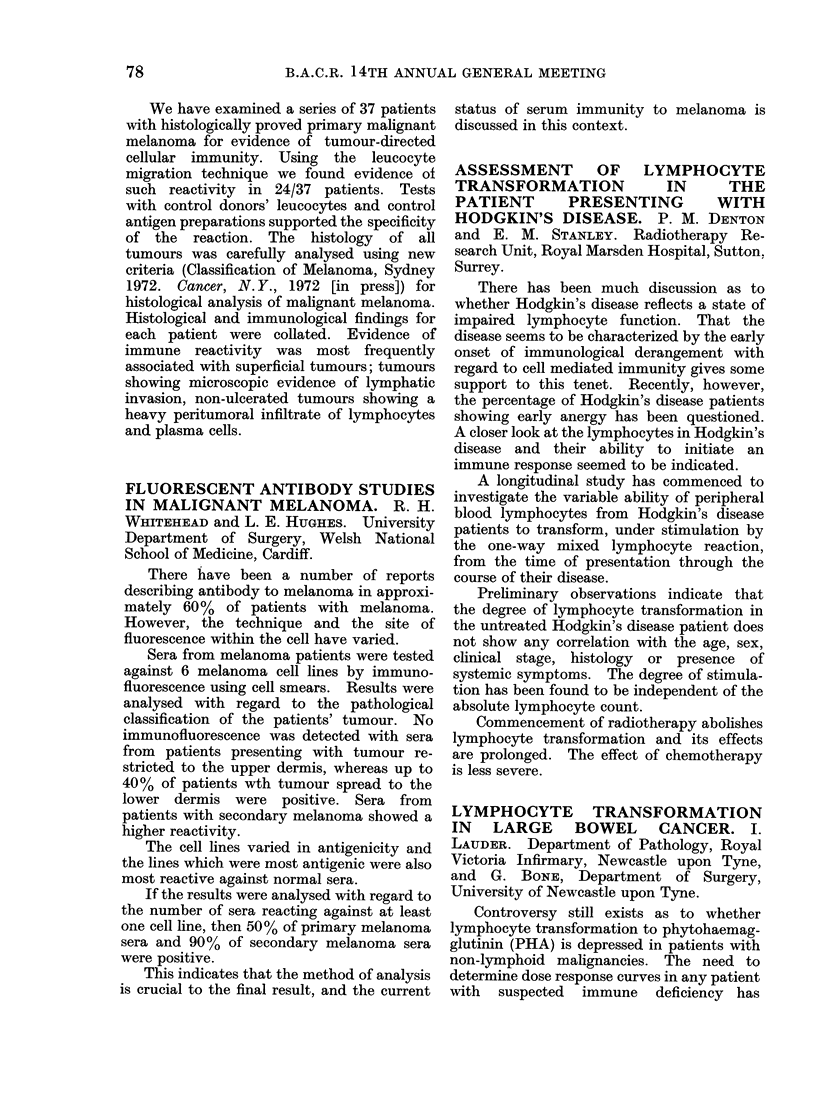# Fluorescent antibody studies in malignant melanoma.

**DOI:** 10.1038/bjc.1973.80

**Published:** 1973-07

**Authors:** R. H. Whitehead, L. E. Hughes


**FLUORESCENT ANTIBODY STUDIES IN MALIGNANT MELANOMA.** R. H. WHITEHEAD and L. E. HUGHES. University Department of Surgery, Welsh National School of Medicine, Cardiff.

There have been a number of reports describing antibody to melanoma in approximately 60% of patients with melanoma. However, the technique and the site of fluorescence within the cell have varied.

Sera from melanoma patients were tested against 6 melanoma cell lines by immunofluorescence using cell smears. Results were analysed with regard to the pathological classification of the patients' tumour. No immunofluorescence was detected with sera from patients presenting with tumour restricted to the upper dermis, whereas up to 40% of patients wth tumour spread to the lower dermis were positive. Sera from patients with secondary melanoma showed a higher reactivity.

The cell lines varied in antigenicity and the lines which were most antigenic were also most reactive against normal sera.

If the results were analysed with regard to the number of sera reacting against at least one cell line, then 50% of primary melanoma sera and 90% of secondary melanoma sera were positive.

This indicates that the method of analysis is crucial to the final result, and the current status of serum immunity to melanoma is discussed in this context.